# Systematic and transparent inclusion of ethical issues and recommendations in clinical practice guidelines: a six-step approach

**DOI:** 10.1186/s13012-014-0184-y

**Published:** 2014-12-04

**Authors:** Marcel Mertz, Daniel Strech

**Affiliations:** Institute for History, Ethics and Philosophy of Medicine, Hannover Medical School, Carl-Neuberg-Str. 1, 30625 Hannover, Germany; Research Unit Ethics, Institute for History and Ethics of Medicine, University Hospital Cologne, Herderstr. 54, 50931 Cologne, Germany

**Keywords:** Clinical practice guideline, Guideline development, Ethical recommendation, Clinical ethics, Medical professionalism

## Abstract

**Background:**

Clinical practice guidelines (CPGs), a core tool to foster medical professionalism, differ widely in whether and how they address disease-specific ethical issues (DSEIs), and current manuals for CPG development are silent on this issue. The implementation of an explicit method faces two core challenges: first, it adds further complexity to CPG development and requires human and financial resources. Second, in contrast to the in-depth treatment of ethical issues that is standard in bioethics, the inclusion of DSEIs in CPGs need to be more pragmatic, reductive, and simplistic, but without rendering the resulting recommendations useless or insufficiently justified. This paper outlines a six-step approach, EthicsGuide, for the systematic and transparent inclusion of ethical issues and recommendations in CPGs.

**Methods:**

The development of EthicsGuide is based on (a) methodological standards in evidence-based CPG development, (b) principles of bioethics, (c) research findings on how DSEIs are currently addressed in CPGs, and (d) findings from two proof-of-concept analyses of the EthicsGuide approach.

**Results:**

The six steps are 1) determine the DSEI spectrum and the need for ethical recommendations; 2) develop statements on which to base ethical recommendations; 3) categorize, classify, condense, and paraphrase the statements; 4) write recommendations in a standard form; 5) validate and justify recommendations, making any necessary modifications; and 6) address consent. All six steps necessarily come into play when including DSEIs in CPGs.

**Conclusions:**

If DSEIs are not explicitly addressed, they are unavoidably dealt with implicitly. We believe that as ethicists gain greater involvement in decision-making about health, personal rights, or economic issues, they should make their methods transparent and replicable by other researchers; and as ethical issues become more widely reflected in CPGs, CPG developers have to learn how to address them in a methodologically adequate way. The approach proposed should serve as a basis for further discussion on how to reach these goals. It breaks open the black box of what ethicists *implicitly* do when they develop recommendations. Further, interdisciplinary discussion and pilot tests are needed to explore the minimal requirements that guarantee a simplified procedure which is still acceptable and does not become mere window dressing.

## Background

Clinical practice guidelines (CPGs) are an internationally established tool for improving quality of clinical practice around specific diseases or clinical symptoms. Increasingly, their development includes measures to strengthen validity and accountability, such as explicit procedures to grade the strength of recommendations [1], to manage conflicts of interest [2], or to increase participation of patient representatives [3]. They are meant to raise standards of clinical competence and medical professionalism by referring explicitly to evidence on benefits and harms [4]. Medical professionalism, in turn, requires awareness and careful handling of disease-specific ethical issues (DSEIs) [5].

However, recent research findings show that CPGs differ widely in whether and how they address general and DSEIs and whether they include any recommendations on how to deal with such DSEIs [6].

How does a DSEI arise? The majority of widely shared frameworks for medical professionalism and common approaches to morality in bioethics are based on a set of prima facie binding ethical principles: respect for patient autonomy, beneficence, non-maleficence, and justice [5,7,8]. Once we accept that these ethical principles are relevant to health-related decision-making, a DSEI can arise from (a) neglect of one or more ethical principles, for example, “Insufficient consideration of patient autonomy and patient preferences in dementia care decisions” [9], or (b) conflicts between two or more ethical principles, for example, “Balancing the do-no-harm principle (non-maleficence) versus the freedom-to-move-at-will principle (patient autonomy) in decision-making for or against physical restraints on account of inappropriate patient behavior” [9]. For further examples of DSEI in dementia care, see [9]. For a demonstration of how current dementia guidelines describe DSEIs, see the supplementary material in [6].

Awareness not only of the four general ethical principles but also especially of relevant DSEIs, and competence in managing these DSEIs, are deeply intertwined with the concepts of clinical competence and professionalism of health care workers [9]. Furthermore, awareness of and competence with DSEIs is important also for third parties, such as relatives or hospital managers.

The inclusion of DSEIs backed up with ethical recommendations should be a priority for the following reasons. First, being unaware of ethically sensitive situations may lead to ethically problematic behavior. Furthermore, lacking guidance for ethically sensitive situations amounts to a failure of the central task of a CPG, namely to give information and orientation in medical practice. The appropriate inclusion of DSEIs, therefore, aims to further improve the quality of CPGs and to make CPGs an adequate tool to foster medical professionalism.

In order to not undermine current standards for CPG development and to generate trust and confidence in ethical recommendations, the writing and appraisal of ethical issues in CPGs should be performed systematically and transparently and with appropriate quality assessment. Unfortunately, there are no established standards or methods for such an enterprise. The current manuals for guideline development from WHO, NICE, the Institute of Medicine (IOM), SIGN, National Health and Medical Research Council (NHMRC), and the Association of Scientific Medical Societies in Germany (AWMF) do not indicate how to address DSEIs [4,10-14]. The field of ethics guideline development also lacks transparent standards and instructions for the formulation and quality control of ethical recommendations [15-18].

When developing a methodology for the systematic, transparent, and—as far as possible—objective (i.e., unbiased, not too dependent on subjective evaluations) inclusion of ethical recommendations in CPGs, some challenges have to be faced. (a) It further complicates CPG development and requires human and financial resources. (b) In contrast with the detailed explanations of ethical issues and justifications of ethical recommendations that are standard among medical ethicists, the inclusion of ethical issues and related recommendations in CPGs must be more pragmatic, reductive, and simplistic. “Pragmatic” means that recommendations should apply directly to the issue at hand, even if some analytical finesse and complexity that would be standard in academic discussions have to be left out; “reductive” means that the justification of a recommendation cannot be built up in a philosophically comprehensive way but has to be narrowed down to a central point in the context of the chosen ethical framework; “simplistic” means that recommendations and their justifications should be comprehensible to practitioners without formal training in medical ethics. (c) While efforts to shorten, simplify, and focus the discussion of ethical issues are important, these efforts if undertaken inappropriately may render any ethical recommendations useless and/or unjustified [19]. An ethical recommendation is “useful” when it provides guidance that can impact clinical practice or decision-making. An ethical recommendation is “justified” when it is clear which normative (principles, norms, values) and possibly empirical reasons show that the recommendation is “morally right” or at least “morally defensible”. Therefore, the inclusion of ethical issues within CPG development needs to meet a minimum standard in the sense of a “level of sufficiency for sound justification and validity” (for further comments on this “level of sufficiency”, see the analysis and discussion sections). The scientific communities of CPG development and medical ethics should work together to develop, evaluate, and continually modify a procedure for the sufficiently sound and nonetheless feasible inclusion of ethical issues and recommendations in CPGs.

In this paper, we propose a six-step approach, called “EthicsGuide”, as a first attempt to address the question of how to integrate DSEIs in CPGs in a transparent and systematic way. EthicsGuide reflects methodological standards for the evidence-based generation of recommendations in CPGs [4] but differs in crucial points, acknowledging the fact that ethical recommendations based on (empirically informed) normative analysis are not of the same type as clinical recommendations based on external and internal evidence.

Because of the abovementioned practical relevance of as well as theoretical challenges to the transparent development of ethical recommendations in CPGs (and elsewhere), our paper addresses two audiences. First, it addresses the guideline development community (including all who contribute to guidelines as health care professionals, patient representatives or health services researchers). For this audience, the paper not only highlights the often implicit methodological steps in the development of ethical recommendations. It also introduces important criteria and standard terminology for assessing the characteristics and the validity of normative analysis relevant to guideline development (e.g., specificity, qualification, degree of obligation, etc.). Second, it addresses the community of medical ethics and bioethics researchers. For this audience, the paper mainly serves as a starting point for discussing the abovementioned need to simplify the procedure while ensuring ethical recommendations are useful and justified.

## Methods

The methodological recommendations made in this paper are the results of our attempt to combine current methodological standards in the fields of (a) normative and empirical bioethics and (b) evidence-based guideline development, based on research that analyzed the status quo of which and how ethical issues are represented in national CPGs for dementia care [6,9]. Basic normative ethical concepts that inform the EthicsGuide approach are given by (a) the abovementioned “four principles approach” [8] and (b) several procedural principles for fair and accountable decision-making, such as systematic conduct, transparency, objectivity, justification, consistency, and feasibility.

Based on prior discussion among all authors, a first version of the technical requirements for the six steps described in this paper was developed. We performed a proof-of-concept analysis for this version by exemplarily developing ethical recommendations for two ethical issues in dementia care; this means, we went through the six steps, focusing especially on step 5, in order to check if it is possible to generate ethical recommendations with EthicsGuide (step 6 was not part of the proof-of-concept analysis, as there was no guideline working group involved). The proof-of-concept analysis informed a revision addressing issues of validity and consistency. A second, shorter proof-of-concept analysis concerning ethical issues in chronic kidney disease was also conducted, which also showed the general feasibility of using the framework to generate ethical recommendations. (In the following, we will use examples from the proof-of-concept analysis for dementia care in order to illustrate various steps; at this moment, other examples for applying the EthicsGuide are not available.)

We assume that ethical recommendations for CPGs are always generated and written by a group of authors, most often the author group responsible for the CPG itself, or more specifically, a sub-group of this author group. In the following, we will use “author group” for both types.

## Results and discussion

We now describe all six steps of the EthicsGuide framework in chronological order.

### Step 1: Determining the DSEI spectrum and the need for ethical recommendations

**Figure 1:**
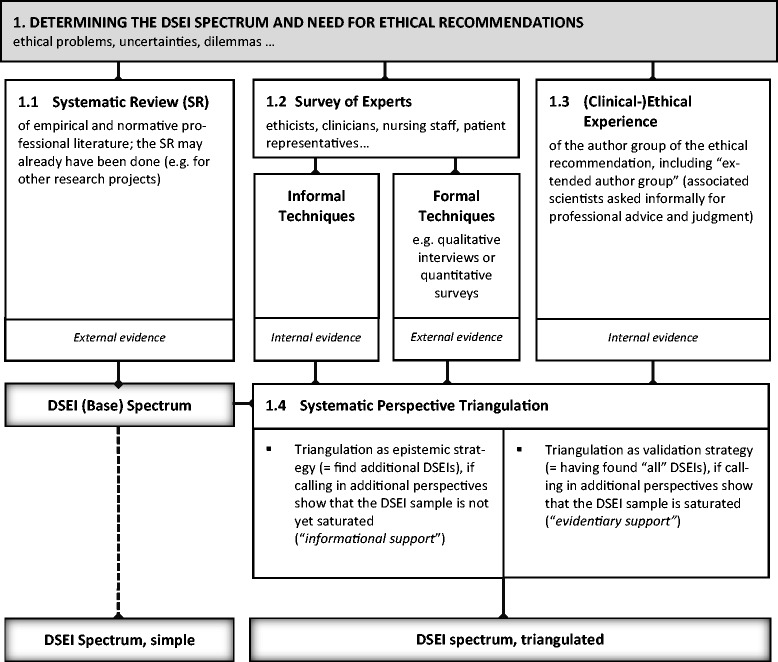
**Visualization of step 1.**

(For a visualization of the following explanation of step 1, see Figure 1).

To be justified in a practical setting, an ethical recommendation should be practically helpful—whether by answering open questions, reducing uncertainty, or giving concrete orientation in situations where it is unclear how to choose between different options.

From our experience of formulating and evaluating ethical recommendations in CPGs [6], we suggest using a systematic review in order to establish a “*full spectrum of DSEI[s]”* [9] to support the CPG development panel in (a) understanding what DSEIs are at stake, (b) collecting statements (descriptive or normative) from the literature about these DSEIs, and (c) prioritizing the DSEIs that are deemed relevant enough to formulate recommendations for the particular CPG.

Building up this spectrum of DSEIs can be done in several ways. The methodologically ideal way would be a multi-method approach synthesizing external and internal sources for relevant empirical and argument-based evidence: (a) a systematic review of empirical *and* argument-based literature about the social and moral context of the specific disease, (b) the expertise of external experts in the relevant fields, and (c) the experience of the author group. This sort of “s*ystematic perspective triangulation*” [20] can be used as a strategy to strengthen the validity of assertions about the *completeness* of the DSEI spectrum when additional perspectives give the same results.

### Step 2: Collecting relevant data and statements for an ethical recommendation

**Figure 2:**
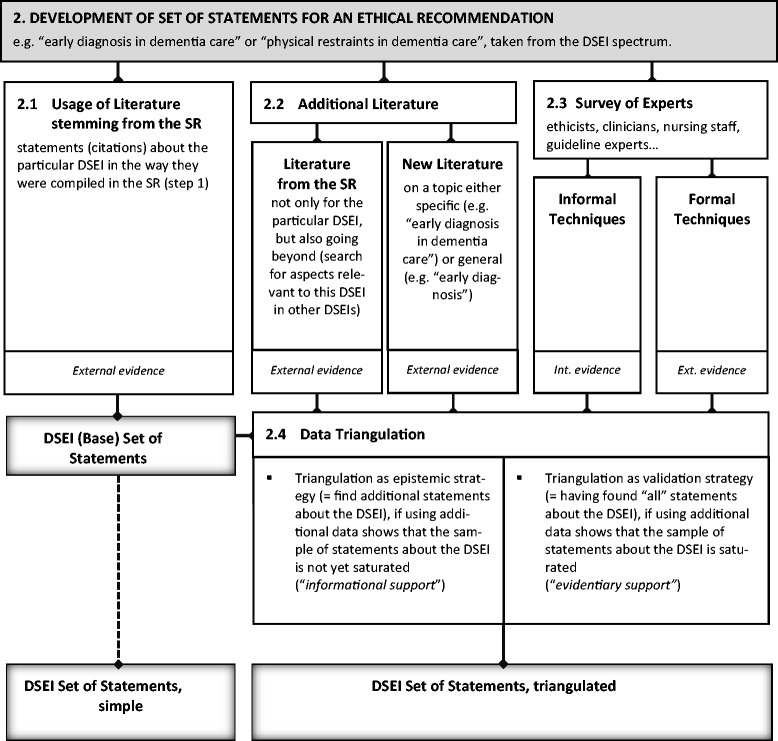
**Visualization of step 2.**

(For a visualization of the following explanation of step 2, see Figure 2).

The goal of the second step is to establish a *set* of relevant empirical data and argument-based *statements* needed to develop explanations and recommendations for each CPG-relevant DSEI. For example, in one of our proof-of-concept analyses, we identified the following two statements (among others) from the literature captured in the systematic review of step 1 that we deemed relevant clinical and normative statements for the DSEI “Dealing with physical restraints”: “Risks associated with use of restraints: Injuries from falls, accidental death from strangulation, decline in function, skin breakdown, cardiac stress, reduced appetite, dehydration, emotional and behavioral problems” [21]; “The fairest approach to striking this balance between safety and independence is to restrict autonomous behavior only in those people who are a danger to themselves and others” [22].

From a pragmatic point of view, the tool of choice for generating this set of relevant empirical and normative statements is the systematic review of DSEIs from step 1. Additional means are further literature searches and again a survey of experts. Obviously, a central problem with searching statements for each DSEI is of a practical nature: how in-depth and inclusive should the search of statements be? How much additional literature should be searched, considering that one may find thousands of hits when using online databases or, for example, Google Books, especially if there is a large literature about a specific disease? Author groups have inevitably to make a pragmatic decision about how to deal with this problem. It should be clear, however, that refraining from any sort of searching of relevant *external* statements as a basis for developing ethical recommendations—and thus relying solely on the knowledge of the author group—goes against the very concept of evidence-based guideline development and takes us back to the concept of “eminence-based” guideline development. It should be carefully considered within the author group whether the latter approach is the only possible one, especially when it comes to national or international guidelines for diseases such as dementia that involve a broad spectrum of DSEIs and a broad spectrum of normative statements on how to deal with these DSEIs. From the experiences of our proof-of-concept analyses, we believe that trained ethicists could skim through the identified literature and extract relevant statements for several DSEIs in a relatively timely manner (untrained persons in the guideline working group may need more time if they are less familiar with ethical terminology and argumentation). For example, it took about 15 to 60 min per peer-reviewed journal article (about 5,000 words), relative to the respective relevant content of the article, to check for statements. This makes about 300 to 1,800 min for 20 to 30 articles. In the shorter proof-of-concept analysis concerning ethical issues in chronic kidney disease, there were fewer articles and less relevant content in the articles. The amount of time needed to transparently develop sets of statements for DSEIs depends on the topic and the current state of the academic discussion in the field.

For examples of methodologically different systematic reviews of normative literature for a specific ethical issue (not “disease specific” in each case), see [23-26]. Again, triangulation can be used to merge different sources of statements about the DSEI in question.

How to determine the quality of descriptive or normative statements about DSEIs is another contentious issue [27,28]. Following the procedural approach to quality assessment, it is possible to determine the quality of the statements on the basis of their generation—systematic review, additional literature search, survey of experts—according to the methodological effort expended.

### Step 3: Categorization/classification and synthesis/paraphrasing of statements

**Figure 3:**
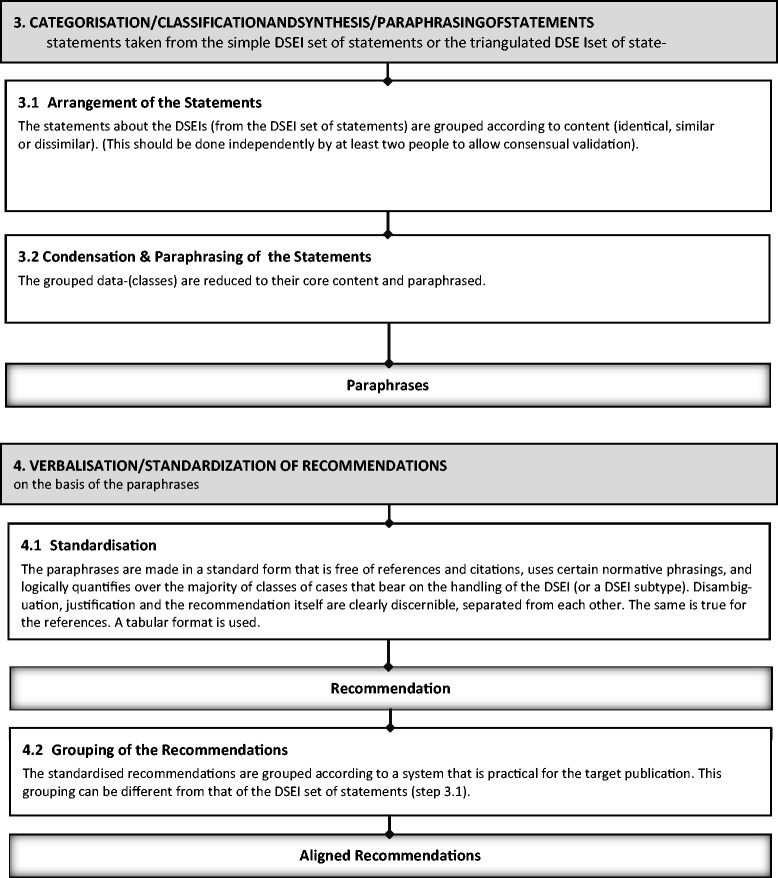
**Visualization of steps 3 and 4.**

(For a visualization of the following explanation of step 3, see Figure 3).

In the following, we assume that at least some part of the empirical and normative statements for each relevant DSEI is derived from systematic approaches taking knowledge from outside the author group into account. Following step 2, statements with identical or similar content have to be distinguished from statements that are dissimilar to each other, and grouped in corresponding sub-categories. In our proof-of-concept analyses, such sub-categories for the DSEI “physical restraints” were, for example, “goals/aims of using restraints”, “justifications of using restraints: risk to others”, “examination of medical and psychological causes for the necessity of using restraints,” and “improvement of general conditions in the handling of patients with dementia (in order to reduce the necessity of using restraints)”, among others. This task is typical for analyzing qualitative text, as in thematic text analysis [29]. For greater validity, at least two researchers should agree to these categorizations.

But even categorized, empirical and normative statements will still be just so much information. In order to make this information manageable and usable for the development and articulation of an ethical recommendation, it must be *synthesized*. Instead of having several statements, for the same or a sufficiently similar statement (core content), a *single statement* has to be worked out. This single statement is, methodologically speaking, a *paraphrase*. In this paraphrase, the core content will be freed from all contingency stemming from its source viz. will be a generalized statement without (a) the style of a particular author, (b) mention of particular institutions, or (c) citations and references. So, for example, in the proof-of-concept analysis, the paraphrase for the category “risks of using restraints: harming interests” was based on two longer statements and formulated as follows (translated from German): “Besides physiological and psychological harms and risks, physical restraints can also harm other interests of a person, such as having a protected area of privacy or the interest of interacting with a psychosocial environment”.

The paraphrasing step could be left out or combined with step 4; but especially when confronted with complex information, paraphrasing as a separate step aids transparency, comprehensibility, and accurateness. Of course, for a single DSEI, there can be several paraphrases with dissimilar content, referring to different ethical aspects of the DSEI.

### Step 4: Verbalization/standardization of recommendations

(For a visualization of the following explanation of step 4, see Figure 3).

In step 4, the subgroup of the guideline development group that is dedicated to ethical issues *standardizes* the paraphrases’ (logical) structure and textual style. In this way, the paraphrased statements produced at the end of step 3 are rephrased. This addresses three concerns: (a) it makes the recommendation immediately identifiable (thus distinguishable from and comparable with other passages in a text), (b) it avoids recommendations that merely summarize or describe the data/statements, and (c) it allows its parts to be separated (the essentially normative part of a recommendation should be separate from its justification). The separation of (the essential) recommendation and its justification (if any) is also important for the presentation in *tabular form* (see Table 1).

**Table 1:** **Examples of tabular form of a recommendation**

	**Two examples of ethical recommendations**
Example 1	
Presupposition	For all cases of the use of physical restraints, it is true that certain elderly people have a higher risk of being subjected to restraints than other elderly people if they exhibit the following traits: functional disabilities, higher dependencies for day-to-day-activities, mobility problems, cognitive disturbances, behavioral problems, or a history of multiple falls
Recommendation	The use of restraints has to be especially justified and reviewed in all cases that apply to the presuppositions
Justification	None given
Elucidations/comments	*Authors:* the stated traits can be interpreted as risk factors for being subjected to physical restraints, irrespective of whether or not they are justified. As elderly people with these risk factors have a higher probability of being subjected to physical restraints that may also be unjustified, special care is judicious as soon as a patient exhibits such traits
References	*“Older people with functional disabilities, increased activities of daily living dependence, mobility problems, cognitive disturbances, behavioral problems, or a history of multiple falls run a much higher risk of being physically restrained.”*
	*(Gastmans C, Milisen K. Use of physical restraint in nursing homes: clinical-ethical considerations. J Med Ethics. 2006 Mar;32(3):148–52,148).*
Example 2	
Presupposition	For all cases of the use of (physical) restraints, it is true that the reasons for applying them may change as situations change
Recommendation	The use and the rationale of (physical) restraints have to be re-evaluated on a regular basis
Justification	This takes place on patient’s behalf:
	(Physical) restraints must not be longer used as necessary for reaching the goals that were initially defined for applying them
	(Principle of Nonmaleficence, Principle of Respect for Autonomy)
Elucidations/comments	For this, the use of physical restraints should be stopped periodically, and the patient should be put under continuous monitoring regarding her/his physical condition (e.g., skin color, extremity movement, sensation) and her/his personal needs (e.g., toileting, food, fluids).
References	*“The proposed ethical guidelines devised by the Ethics and Humanities Subcommittee of the American Academy of Neurology include the following:*
	*1.) restraints should be ordered when they contribute to the safety of the patient or others and are not simply a convenience for the staff*
	*2.) restraints should not be ordered as a substitute for careful evaluation and surveillance of the patient, as appropriate for good medical practice*
	*3.) the perceived need for restraints should trigger medical assessment and investigation of the precise reason for them, intended to correct the underlying medical or psychological problem*
	*4.) if a proxy decision maker is known, restraints should be ordered after full discussion of the risks and benefits. However, in an acute situation doctors should act in the best interest of the patient*
	*5.) when they are indicated, pharmacological agents should be used at the lowest dose possible*
	*6.) all restraints should be reassessed frequently so that they may be in effect for the shortest duration necessary to achieve their goals”*
	*(Rai, Guchuran S/Eccles, Jim, Ethical issues in dementia, in: Rai, Gurchuran S., Medical Ethics and the Elderly, Oxford 2009, 125–137, 133).*
	*“[…] For example, Moss and La Puma (1991) and Evans and Strumpf (1989) proposed the following ethical guidelines: (1) mechanical restraints should never be ordered routinely or as a substitute for careful patient surveillance; (2) orders for restraints should trigger a medical investigation aimed at identifying and correcting the medical or psychological problem responsible for the order; (3) the patient’s surrogate decision-maker should consent to the restraints and be given full disclosure of the risks and benefits; (4) when indicated, mechanical restraints should be applied carefully, temporarily, and with the least-restrictive device possible; and (5) when indicated, pharmacological restraints should be prescribed with the proper agent in the lowest effective dose and with frequent reassessments.”*
	*(Bernat, Jamels L, Ethical Issues in the care of the patient with dementia, in: Duyckaerts C, Litvan I (eds) Handbook of Clinical Neurology, Vol. 89 (3rd series) Dementias, 115–130, 121–122).*
	[There are three further references that, for readability reasons, are not included in this example]

In the course of the standardization procedure, the *degree of obligation* should be made clear. This is achieved by defining a hierarchy of *normative functors* (such as “should”, “must”, “must not”, “may”) as is standard practice in present guideline development. While the hierarchy of *normative functors* has construct validity, it is important to acknowledge that this concept does not always work as intended in practice. Research findings within the CPG development community, for example, suggests that guideline users do not necessarily perceive recommendation strengths as the guideline authors intended [30].

It is important to acknowledge that for reasons of reproducibility and transparency, no modifications or additions to the “external set of statements” are allowed up to this point (end of step 4). As a consequence, the author group should embrace a certain attitude of discipline in order to safeguard the traceability of the generation process of a recommendation and, thus, wait until step 5 before modifying the externally derived statements and their resulting paraphrases and condensations to address quality issues—even if it is foreseeable at an earlier step that a recommendation cannot be put forward in its original form. We will return to this challenge in the discussion section.

It proved advantageous in our proof-of-concept analyses to use a tabular form to display the recommendations. Tabulations can provide more clarity than continuous text and should be used at least when constructing recommendations; the final formatting of the recommendations will also depend on the publication format. In tabular form, the topmost line is reserved for the *recommendation* itself, followed by the *justification* of the recommendation in the next line. If there is no justification available in the paraphrases, it can be added in step 5. *Elucidations* and *comments* are reserved for the next line; if these are added by the authors (and not part of the paraphrases), they must be declared as such. The bottommost line contains the *references*, that is, the original citations from which the recommendation has been condensed and paraphrased, with a complete indication of its source. See Table 1 for an example of a recommendation in tabular form derived from one of the proof-of-concept analyses.

The condensation and paraphrasing of statements to obtain a recommendation is *only* justified by *inductive generalization*, as it is a consequence of the standardization that singular statements (the paraphrase) are generalized. Also, the claim that the recommendation is true for most cases is generalized from the external statements available. Note that the *normative* justification of a recommendation—its validity or rightness from a normative ethical point of view—is *not* based on this inductive generalization. Further, a fifth step is needed to supply normative justification.

### Step 5: Validation, modification, and justification of a recommendation

**Figure 4:**
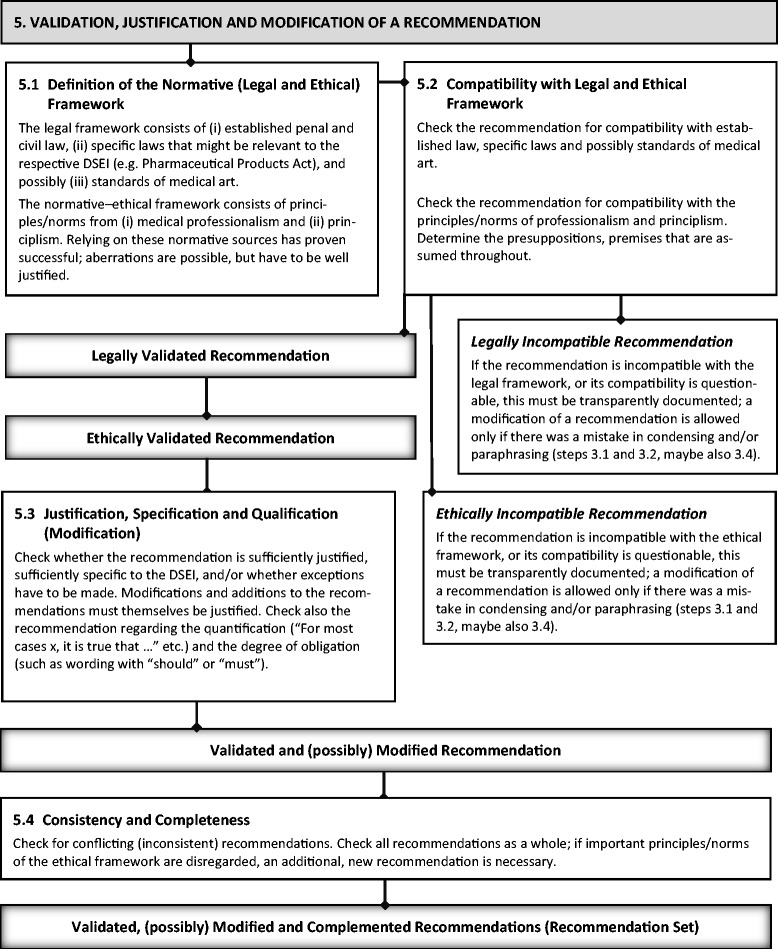
**Visualization of step 5.**

(For a visualization of the following explanation of step 5, see Figure 4).

Up to this point, the development of standardized recommendations derived from a set of external statements (DSEI statement set) as found in the literature (steps 1–4) is mainly, though not exclusively, a descriptive process. As paraphrasing statements and standardizing recommendations include interpretation and judgments of relevance and order, there is *implicit normativity*, but it should be made explicit enough by the transparent and documented generation of the recommendations.

But even if there is already some normativity, it is not the normativity sought in order to make sure that the recommendation is actually *valid*. It would be methodologically and also ethically problematic to compile recommendations from statements without building in steps to ensure quality from an explicit normative point of view. What is needed to *validate* the recommendations is a normative evaluation from a legal and ethical perspective. Researchers charged with validation need sufficient expertise with the relevant legal systems and a certain sensibility for normative ethical work (e.g., an understanding of using principles for argumentatively justifiying or criticizing normative statements). For reasons of brevity, we refrain from describing the professional procedure of how an expert in law will check the legal compatibility of a certain recommendation. Nevertheless, the results of the legal evaluation should also be documented transparently.

The ethical evaluation must first define the relevant normative framework. This entails determining ethical principles (ethical norms) that are used for validating recommendations. We propose that author groups for CPGs refer to principles or codices from *medical professionalism* frameworks [5], which are often based on the ethical approach of *principlism* (“four-principles approach”, see the “Methods” section above) [8]. Deviations or modifications of these broadly accepted ethical frameworks are possible, but should be justified (why one does not find those broadly accepted principles/codices appropriate or why one finds the addition of other principles or norms crucial etc.).

Afterwards, *validation* (or ethical justifiability/acceptability) is sought by evaluating recommendations for *compatibility with the ethical framework*. Again, this evaluation should be documented transparently, e.g., by naming the principles or codices used and recording whether the recommendation is compatible with the said principles or codices, and if not, why it is not compatible. In our proof-of-concept analysis, for example, we used ten principles of medical professionalism [5], the nine principles of the “standard of conduct” from the APA [7], and the four principles of principlism [8]. In each recommendation, we recorded whether it is compatible with these sets of principles, and if yes, just added a “compatible” remark; otherwise, we identified the concrete principle and briefly noted the conflict.

This step aims to prevent is ought fallacies in the generation of a recommendation (“ought” statement) from a descriptive compilation (“is” statement) of the DSEI set of statements.

To preserve transparency and traceability as the (external) ethical recommendations derived in steps 1–4 are modified by the authors group, the validation process (in step 5) should be separated from later modification of the recommendation arising from adjustment to the legal or ethical framework. This again might differ from the current practice of bioethicists, who are habituated (mostly for pragmatic reasons of efficiency) to combine the steps of (a) validation and (b) modification of ought statements (ethical recommendations). To preserve traceability, any modification of a recommendation up to the step of validation should only be accepted if there was a “technical” mistake, for example, in condensing and/or paraphrasing (steps 3 and 4). Otherwise, it will be hard to accurately criticize the author group’s judgements in the development of ethical recommendations, to say for example “I would have grouped it in another way”, “Here, the paraphrase seems problematic”, “Additional normative–ethical conditions are needed”.

When the validation step is complete, the modification of a recommendation can begin. This step might include the modification of an already given justification for a recommendation to improve its argumentative correctness and consistency. In some cases, the author group might need to add a completely new justification. All modifications and inclusions of new content should be explicitly highlighted. Justifications should *at least* make clear from which principles of the chosen ethical framework the recommendation gains its normative power.

Some of the following strategies/methods/tools often play an important role in the improvement of justifications for ethical recommendations: (a) *Specifications:* is it necessary to further specify the recommendation to the respective DSEI? (b) *Qualifications*: is it necessary to state important exceptions where the recommendation does not hold true? (c) *Balancing*: how can *conflicts between principles* be solved (balanced)? Balancing becomes important when conflicts of principle cannot be solved by specifications or qualifications.

For example, in the proof-of-concept analyses, we had to add a specification to the following recommendation: “For most cases when using restraints, it is true that restraints should be used when the patient is no longer capable of making informed decisions, but the care team is considering the medical or nursing intervention as being in the interest of the patient (‘best interest’)”. The specification reads as follows: “[…] as being in the interest of the patient (‘best interest’), and there is no feasible option available to gain proxy consent from e.g. relatives of the patient.” This was relevant because with this specification, possible conflicts with principles of the codices of the APA (respect for the rights of others) and principlism (respect for patient autonomy) could be solved. The specification and its justification were explicitly added in the tabular form of the recommendation.

On the basis of the (modified or added) justification, it might be essential to adjust the quantification as well the degree of obligation. As specification, qualifications, balancing, and quantifications, all necessarily involve modification by the author group of the externally derived set of normative statements, they should be made transparent by explicit documentation. Last but not least, it should be checked whether any recommendations *contradict* each other (are *inconsistent* with each other).

An open question the author group has to tangle with is the level of detail in the justifications. Is a reference to a principle sufficient, or should it be more specific or exact? It is paramount for the group to distinguish (a) theoretical desiderata and (b) practical requirements. Often they will conflict, as from a theoretical point of view, a justification should comply with philosophical-ethical standards, while from a practical point of view, a justification should be understandable by lay people, and may not exceed a certain length. Once more, the author group should take the target publication, its structure and its readership into consideration in order to decide what constitutes a “sufficient” level of detail of justification.

### Step 6: Consenting the recommendations

**Figure 5:**
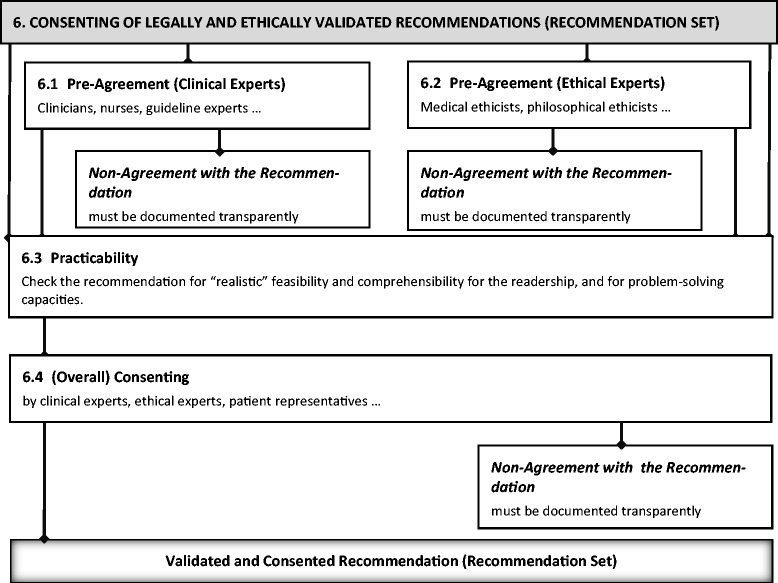
**Visualization of step 6.**

(For a visualization of the following explanation of step 6, see Figure 5).

All final recommendations should be agreed by the CPG development panel. As methods for obtaining consent are already established in the practice of CPG development, we will only give a sketch of this part of the method and focus on particular sub-steps we included specially.

The CPG panel (especially health care experts and patient representatives) should vet the DSEI recommendations for clinical relevance. The CPG panel (especially ethical experts used to discuss and analyze ethical issues in health care) should pre-agree the DSEI recommendation regarding its ethical justifiability. The CPG panel should check the recommendation for *practicability*. This means that the recommendation is agreed to be “realistic” and “useful” (for problem solving) considering the relevant institutional setting etc., and that the intended users will be able to understand the recommendation.

## Conclusions

EthicsGuide is an attempt to give a consistent procedure for a “systematic, objective and transparent development of recommendations for disease-specific ethical issues (DSEIs)”. We are (and everyone reading this text should be) well aware that following this procedure to the letter would require considerable time and skill in normative analysis. Therefore, the description of the proposed method should be understood as a methodological ideal: we claim that each step explicitly addresses a point in the inclusion of ethical issues and recommendations in CPGs which must otherwise unavoidably be addressed in an implicit, opaque way. We acknowledge that there might be good reasons to be more or less transparent about these methodological steps, depending on the aims and the scope of a given CPG and for pragmatic reasons. It is also possible to skip or modify individual sub-steps; but this should be made clear in the process, along with the rationale for skipping them. This is necessary to ensure the possibility of quality assessment of the process. Therefore, it is important that guideline development groups who decide to include DSEIs and recommendations in a more implicit way are aware of the methodological steps and determine how much pragmatism, reduction, and simplification is still appropriate to reach the abovementioned level of sufficiency.

While being aware of the importance of determining objective criteria for the best possible assessment of such a level of sufficiency, we cannot analyze such criteria in this paper because we lack sufficient evidence from more than two proof-of-concept analyses (on two ethical issues in dementia care) that would be necessary to evaluate the validity of such criteria.

The primary value of the ideal procedure described is to function as a starting point for both the medical ethics community and the guideline development community, to (a) have a sound discussion on how to develop ethical recommendations for CPGs, (b) develop procedures that are simpler and more pragmatic, and (c) discuss the minimal requirements that guarantee a simplified procedure is still acceptable and does not become invalid or mere “window dressing”.

While the method described should substantially correspond with what ethicists *implicitly* do (or should do) when they analyze and develop recommendations, the crucial point here is the fact that this is done *explicitly and transparently.* We believe that the more ethicists are involved in decision-making with consequences for health, personal rights, and economic issues, the more they should make their methods transparent and replicable by other researchers; and as ethical issues become more reflected in CPGs, CPG developers have to learn how to address them in a methodologically adequate way. As a comparative analysis of national dementia guidelines, [6] showed that there are considerable differences in the way DSEIs are addressed (if at all), which DSEIs are addressed and to what extent recommendations for them are included; this is also important from an international point of view, as it complicates comparisons between different guidelines and their development strategies. The approach proposed in this paper should serve as a basis for further discussion on how to reach such goals.

A possible further step for EthicsGuide could be to grade ethical recommendations by using scores, analogous (but not identical) to the grading of clinical recommendations. The grading should comprise, we suggest, two separate gradings: the grading of the descriptive and normative validity of the recommendation, and—based on this grading—the grading of the “advisory power” a particular recommendation has (e.g., is it taken as binding, or is it more to be understood as a piece of advice where deviations are not seen as problematic). The grading of descriptive validity would give scores for the degree of descriptive support the underlying DSEI has (see steps 1 and 2), while the grading for normative validity should be separated into normative legal support, normative ethical support, and decisional or consensual support (consenting) (see steps 5 and 6). Perhaps the scores could be combined into a grading scale for “advisory power”.

So, while it is not yet possible to deliver tested and refined scores and gradings for ethical recommendations, we think that it is still important to stress that the abovementioned dimensions of an ethical recommendation *could* be scored and graded in order to improve quality assessment and information regarding ethical recommendations in CPG. Further research and practical experimentation with this regard is required.
